# Hemodynamic rescue after blast injury: intravenous administration outperforms intramuscular vasopressin in a swine model

**DOI:** 10.1007/s00068-026-03155-y

**Published:** 2026-03-24

**Authors:** Mattias Renberg, Jenny Gustavsson, Marius Rehn, Mattias Günther

**Affiliations:** 1 Department of Clinical Science and Education Södersjukhuset, Section for Anesthesiology and Intensive Care, Karolinska Institutet, Sjukhusbacken 10, Stockholm, SE-118 83 Sweden; 2 Department of Neuroscience, Karolinska Institutet, Stockholm, Sweden; 3 Air Ambulance Department, Division of Prehospital Services, Oslo University Hospital, Oslo, Norway; 4 Institute of Clinical Medicine, University of Oslo, Oslo, Norway; 5 Department of Research and Development, Norwegian Air Ambulance Foundation, Oslo, Norway

**Keywords:** Trauma, Blast, Hemorrhagic shock, Hemorrhage, Vasopressin, Intramuscular

## Abstract

**Background:**

Hemorrhage remains the leading preventable cause of trauma mortality. Blast trauma combines primary blast effects with complex secondary and tertiary injuries, precipitating profound hemodynamic instability and microcirculatory disruption. While intramuscular (IM) arginine vasopressin (AVP) stabilizes arterial pressure in isolated hemorrhagic shock, vasopressin’s efficacy in blast-associated hemorrhagic shock is unknown.

**Methods:**

In a randomized, blinded study, 22 swine underwent femoral blast injury and controlled class II hemorrhage. Animals received IM AVP 40 U (two doses 60 min apart due to short half-life and transient effects; *n* = 5), IM terlipressin 2 mg (*n* = 5), intravenous (IV) terlipressin 2 mg (*n* = 5), or saline control (*n* = 7). All subjects received a 500 mL autologous whole blood transfusion and were observed for 120 min. The primary outcome was systolic arterial pressure (SAP). Secondary outcomes included systemic vascular resistance index (SVRI), cardiopulmonary and metabolic variables, and serum AVP to assess IM uptake.

**Results:**

IV terlipressin rapidly stabilized hemodynamics, increasing SAP (mean difference 24 mmHg, *p* = 0.01) and SVRI (mean difference 44593 dynes·s·cm⁻⁵·kg, *p* = 0.004) versus controls, and increased mixed venous oxygen saturation and urine output. Respiratory and metabolic variables were similar across groups. Neither IM AVP nor IM terlipressin generated a meaningful pressor response. IM AVP absorption was profoundly variable and did not reliably translate into hemodynamic stabilization; only one animal demonstrated both high systemic uptake and a sustained response.

**Conclusions:**

In blast-associated hemorrhagic shock, IV terlipressin provided consistent hemodynamic stabilization, whereas IM vasopressin analogues were unreliable, highlighting a critical limitation of the IM route in blast pathophysiology.

**Registry:**

Preclinicaltrials.eu, PCT ID: PCTE0000548, Registration date: 30 October 2024.

## Introduction

Trauma is a leading global cause of death and disability, with hemorrhage representing the most preventable cause of mortality in both civilian and military contexts [1, 2]. Early resuscitation is critical to survival [3], yet in austere or battlefield conditions, access to blood products and advanced trauma care is often delayed. Bridging therapies that rapidly stabilize hemodynamics are therefore essential to extend the window for definitive care.

Arginine vasopressin (AVP) is an endogenous vasoconstrictor released during hypotension; however, depletion can occur during prolonged shock [4, 5]. Exogenous AVP has been associated with improved hemodynamics, reduced transfusion requirements, and enhanced survival in experimental and clinical trauma settings [6, 7]. Importantly, intramuscular (IM) AVP has stabilized hemodynamics and improved cerebral perfusion in hemorrhagic shock models [8, 9], and longer-acting analogues such as terlipressin may provide sustained V_1_-mediated vasoconstriction with potential renoprotective effects [5, 8, 9]. From an operational perspective, IM administration offers clear advantages in prehospital and tactical field care where vascular access may be difficult and monitoring limited [10]. However, its reliability is challenged during severe hemorrhage. Compensatory sympathetic activation induces peripheral vasoconstriction and skeletal muscle hypoperfusion, which, together with hemorrhage-associated microcirculatory disruptions [11, 12], can result in delayed or minimal absorption of IM-administered therapeutics.

Blast mechanisms continue to dominate modern combat [13], accounting for 77.3% of reported injuries among U.S. service members between 2007 and 2017 [14]. This trend has intensified in contemporary warfare. Recent data from the conflict in Ukraine indicate that explosive trauma causes 87.2% of military casualties and an overwhelming 96% of civilian injuries [15, 16]. Consequently, optimized field therapies that remain reliable in this context are urgently needed. Although exsanguination remains the leading preventable cause of death in blast casualties, the underlying pathophysiology differs from simple hemorrhage [1, 17]. Blast trauma combines primary overpressure effects with secondary penetrating, tertiary blunt injuries, quaternary burn and psychological injuries, and quinary toxicological and systemic inflammatory complications [17], producing a complex phenotype of shock. Beyond blood loss, blast can trigger vagally mediated hypotension, microcirculatory disruption, oxidative and nitrative stress, endothelial dysfunction, and receptor hyporesponsiveness, processes that may fundamentally alter drug absorption, kinetics, and pharmacodynamics [18–25].

We previously demonstrated that IM AVP is systemically absorbed and effective in isolated hemorrhage [26]. However, whether these effects translate to the more complex physiology of blast-associated hemorrhagic shock is unknown. Therefore, we compared IM AVP, IM terlipressin, and intravenous (IV) terlipressin in a porcine model of blast trauma with controlled hemorrhage, using systolic arterial pressure (SAP) as the primary outcome. We hypothesized that IV terlipressin would provide superior hemodynamic stabilization compared with IM vasopressin analogues, which may show variable efficacy due to blast-induced pathophysiology.

## Materials and methods

### Study design and ethical approval

This was a randomized, blinded experimental trial approved by the Swedish Board of Agriculture (dnr: 12578 − 2020 and dnr: 8209 − 2025). The manuscript was prepared in accordance with the ARRIVE guidelines. Twenty-two healthy, male, castrated swine (approximately 3 months old) of crossbred Yorkshire/Swedish Landrace (63.2 ± 4.7 kg [mean ± SD]) were subjected to a prehospital, polytrauma model combining a femoral blast injury with controlled hemorrhage, targeting an SAP of 65 mmHg resulting in a Class II hemorrhage. The animals were randomized to receive either two doses of 40 U (2 mL) of AVP IM (Empressin, Orpha-Devel Handels und Vertriebs GmbH, Purkersdorf, Austria) (*n* = 5), 2 mg (17 mL) of terlipressin IM (Glypressin, Ferring Läkemedel, Malmö, Sweden) (*n* = 5), 2 mg (17 mL) of terlipressin IV (Glypressin, Ferring Läkemedel, Malmö, Sweden) (*n* = 5), or an equivalent volume of 0.9% NaCl IV or IM (*n* = 7). All animals received a 500 mL autologous whole blood transfusion. The IM AVP and IV terlipressin arms were introduced via a protocol amendment prior to data analysis. The IM terlipressin dose was 2.2-fold higher than the demonstrated effective IV dose in uncontrolled hemorrhage models [8]. To isolate the impact of administration route from drug potency, the IV terlipressin group received an equivalent dose. An IV AVP group was not included because its short biological half-life renders a single bolus insufficient, and continuous infusions are highly impractical in austere prehospital settings. The study was preregistered at preclinicaltrials.eu, PCT ID PCTE0000548.

The primary outcome was SAP at the end of the whole blood transfusion. Secondary outcomes were hemodynamic and respiratory parameters.

**Fig. 1:**
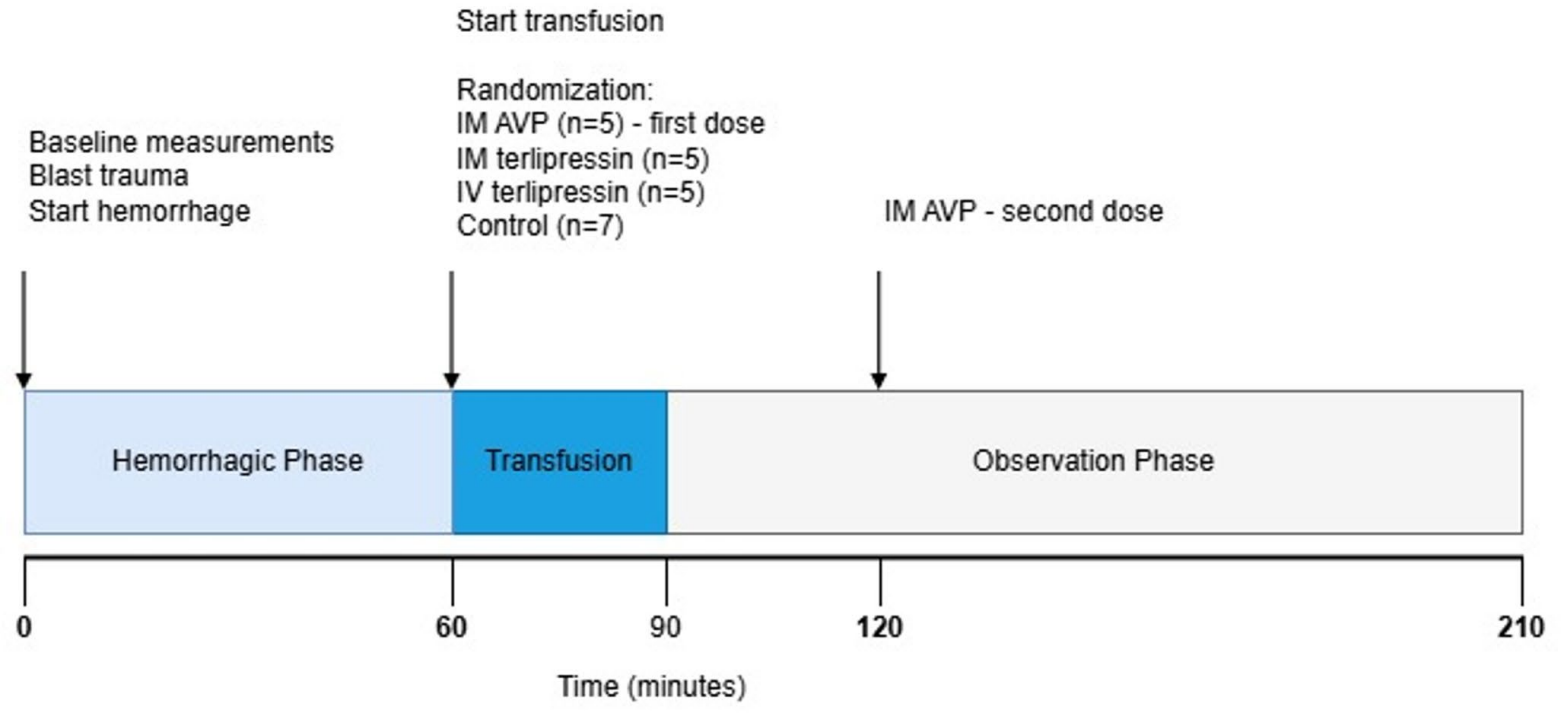
Experimental Timeline and Study Protocol. After baseline measurements, an initial blast trauma was induced, immediately followed by initiation of controlled hemorrhage (T = 0), marking the start of the 60-minute hemorrhagic phase. At T = 60, resuscitation with whole blood transfusion began, and animals were randomized into four intervention groups: IM AVP (*n* = 5), IM terlipressin (*n* = 5), IV terlipressin (*n* = 5), or a saline Control (*n* = 7). The transfusion phase lasted until T = 90, followed by a 120-minute observation phase. The IM AVP group received a second dose at T = 120. The experiment concluded at T = 210. Blood samples were drawn at T = 0, 60, 120, and 210. For animals that succumbed to trauma before the end of the observation period, terminal blood samples were drawn at the time of death. Abbreviations: AVP, arginine vasopressin; IM, intramuscular; IV, intravenous

### Experimental protocol

The experimental design is shown in Fig. 1. A blast injury was induced over the left distal femur as previously described [27], using Swedish military-grade plastic explosive (M/46). M/46 consists of 86% penthyl (pentaerythritol tetranitrate) and 14% mineral oil, yielding a detonation velocity of 8400 m/s. A 2-gram charge of M/46 was placed directly over the distal part of the femur. The animals were covered with ceramic plates from military-grade body armor to direct the blast wave. The charge was taped to a non-electric initiation system capsule and an SL-0 shock tube cord, which was triggered using a Nonel Dynostart ignition box (Dyno Nobel, Brisbane, Australia). This targeted detonation resulted in a severe extremity trauma, characterized by massive avulsion of the skin and underlying musculature, disruption of major vascular structures, including arterial lacerations, and multiple complex open fractures of the femur (corresponding to an Abbreviated Injury Scale severity of 4 = severe). Immediately following the blast injury, a controlled hemorrhage was performed over 60 min using a peristaltic pump (Masterflex L/S, Cole Parmer, Vernon Hills, Illinois). Bleeding was titrated to a target SAP of 65 mmHg by the end of the hemorrhagic phase to mimic a severe, hemorrhagic shock, which resulted in a Class II hemorrhage. Because the profound systemic stress inherent to blast trauma synergistically amplifies hemodynamic instability [28], a Class II hemorrhage in this model reliably precipitates a severe state of shock comparable to that observed with larger-volume hemorrhage in isolated hemorrhage models. The hemorrhage was temporarily paused at an SAP of 75 mmHg to allow hemodynamic evaluation. Shed blood was collected in citrated bags (Fresenius Kabi, Uppsala, Sweden) and stored at room temperature. Total blood volume was estimated at 67 mL/kg.

After the 60-minute hemorrhage phase, the animals were randomized into their respective groups and received either an IM injection in the right lateral thigh, contralateral to the blast injury, or an IV injection. The injections were prepared in unmarked syringes by an unblinded assistant using either 2 mL of refrigerated Empressin (Orpha-Devel Handels und Vertriebs GmbH, Purkersdorf, Austria), 17 mL of refrigerated terlipressin (Glypressin, Ferring Läkemedel, Malmö, Sweden), or 2 mL or 17 mL of refrigerated 0.9% NaCl. Intramuscular AVP was administered at the end of the hemorrhagic phase, with an additional dose given 60 min later. Terlipressin was administered either intramuscularly or intravenously as a single dose at the end of the hemorrhagic phase, with the IM dose given in fractions of 5 mL [29].

Control animals receiving NaCl were randomized to receive either 2 mL at the end of the hemorrhagic phase and 60 min later, or 17 mL IM divided into 5 mL doses, or 17 mL IV. All injections were performed by the same investigator (M.R.) to minimize variability, and injection sites were monitored throughout the experiment for adverse reactions. Immediately after the intervention, all animals received a 500 mL autologous whole blood transfusion over 30 min, followed by 120 min of observation. If glucose levels were below 4.0 mmol/L, the animals received a 30 mL IV bolus of 30% glucose. Surviving animals were euthanized with Pentobarbital sodium (Allfatal vet, 100 mg/mL, Omnidea AB, Stockholm, Sweden) at a dose of 1 mL per kilogram.

### Animal preparation and anaesthesia

Prior to inclusion, the animals were housed for a minimum of 5 days for acclimatization in accordance with institutional guidelines, in an accredited facility with unrestricted access to water and a 12-hour dark/light cycle with 30-minute transition periods. Premedication was administered IM using a combination of Tilatemin/Zolazepam (Zoletil, Virbac, Carros, France) 50 mg/mL and Medetomidin (Cepetor vet, VM Pharma, Stockholm, Sweden) 1 mg/mL (total of 3.8 mL). Bilateral auricular vein cannulation provided IV access. After approximately 3 min of preoxygenation with 100% oxygen, anesthesia was induced with Pentobarbital (Pentobarbitalnatrium vet. APL, Apotek Produktion och Laboratorier, Stockholm, Sweden) 6 mg/kg and Fentanyl (Fentanyl B.Braun, B.Braun Medical AB, Melsungen, Germany) 2.5 µg/kg. Endotracheal intubation was performed with a custom-made Miller blade and a size 8 cuffed endotracheal tube, and ventilation was managed with a Hamilton C2 ventilator (Hamilton Medical, Geneva, Switzerland) in pressure-controlled mode. Ventilatory parameters were set to maintain normal ventilation (pCO₂ 4.7–5.7 kPa) at baseline, with a positive end-expiratory pressure of 5 cmH₂O, fraction of inspired oxygen of 0.21, and tidal volumes of approximately 8–10 mL/kg of body weight. Alveolar recruitment was performed after induction. All animals received a 500 mL bolus of Ringer’s acetate to minimize potential fluid balance differences. No maintenance fluids were given during the experiment. Anesthesia was maintained using continuous infusions of the hemodynamically stable agents ketamine (Ketabel vet., Orion Pharma, Esbo, Finland) at 25 mg/kg/h and midazolam (Midazolam hameln, Hameln Pharma, Hameln, Germany) at 0.0485 mg/kg/h, with analgesia provided by fentanyl (Fentanyl B.Braun, B.Braun Medical AB, Melsungen, Germany) at 3.5 µg/kg/h. Anesthetic depth was assessed continuously utilizing a multimodal approach, combining nociceptive hoof withdrawal reflex testing with the continuous evaluation of vital parameters and autonomic responses. The animals were actively cooled with ice packs when their core body temperature reached 40.5˚C. This intervention threshold was chosen to prevent dangerously high stress-induced temperatures and to ensure the reliability of thermodilution-based cardiac output measurements. Cooling was intentionally discontinued once the temperature fell to 40.0˚C to prevent subsequent iatrogenic hypothermia.

### Monitoring and instrumentation

Arterial lines were placed in the right brachial and femoral arteries to enable continuous pressure monitoring, blood gas sampling, and controlled hemorrhage. A 12-lead electrocardiogram was applied, and pulse oximetry probes were placed on the tail and snout to monitor oxygen saturation. Urinary output was measured via suprapubic catheter placement. A 7.5 F pulmonary artery catheter (Edwards Lifesciences) was inserted through the surgically exposed right external jugular vein to measure core temperature, cardiac output (CO), mixed venous oxygen saturation, and central venous pressure using a Vigilance II monitor (Edwards Lifesciences, Irvine, California).

## Data collection and laboratory analysis

Baseline measurements were taken before the experiment began. During the study, hemodynamic, respiratory, and metabolic parameters were recorded every 15 min. Arterial blood gas analysis included pH, pCO₂, pO₂, SaO₂, base excess, HCO₃⁻, K⁺, Na⁺, Ca²⁺, glucose, lactate, and hematocrit using a GEM 5000 analyzer (Instrumentation Laboratories, Lexington, Massachusetts). Blood samples for pharmacokinetic and biomarker sampling were collected at baseline, prior to the intervention, and at 60 and 120 min after transfusion. Blood samples were collected at the time of death if it occurred before the end of the experiment.

### Calculations

Oxygen delivery was calculated as: DO₂ = CO × CaO₂; CaO₂ = (1.34 × hemoglobin × [SaO₂/100] + [0.225 × PaO2, kPa]), where CaO₂ represents arterial oxygen content. Oxygen consumption was determined using VO₂ = CO × (CaO₂ - CvO₂), where CvO₂ is mixed venous oxygen content. The oxygen extraction ratio was calculated as oxygen consumption/oxygen delivery. CI, systemic vascular resistance index (SVRI), and oxygen delivery index were indexed to bodyweight (kg). Pulmonary shunt was estimated using venous admixture and the Berggren equation: [30] Qs/Qt = (CcO₂ - CaO₂)/(CcO₂ - CvO₂), where Qs/Qt represents shunt fraction, CcO₂ is pulmonary end-capillary oxygen content (assuming ideal alveolar oxygenation), CaO₂ is arterial oxygen content (SaO₂ × hemoglobin × 1.3), and CvO₂ is mixed venous oxygen content (mixed venous oxygen saturation × hemoglobin × 1.3). The pulmonary vascular resistance index (PVRI) was calculated as (mean pulmonary arterial pressure – wedge pressure)/CO x 80 x kg. SVRI was calculated as ((MAP – central venous pressure)/CO) x 80 x kg.

### Pharmacokinetic and biomarker analysis

Serum concentrations of exogenous AVP and endogenous vasopressin-neurophysin 2-copeptin (vasopressin-NIIC), the precursor of the porcine vasopressin analog lysine vasopressin, were measured to assess systemic drug uptake. After 30 min of incubation at room temperature, serum samples were centrifuged, aliquoted, and stored at -74 °C. AVP analysis was performed on undiluted samples using an enzyme-linked immunosorbent assay according to the manufacturer’s protocols (Arg8-Vasopressin enzyme-linked immunosorbent assay kit, Arbor Assays, catalogue no: K049-H1, Ann Arbor, Michigan). Vasopressin-NIIC was analyzed using enzyme-linked immunosorbent assay per manufacturer instructions (Abbexa Ltd, catalogue no: abx512712, Cambridge, United Kingdom) with sample dilutions of 1:50 and 1:100. No analysis of serum concentrations of terlipressin was available.

### Statistical analysis

All statistical analyses were performed using GraphPad Prism v.10.2 (GraphPad Software, Boston, Massachusetts). Block randomization was performed using https://www.randomizer.org/ for each 5-day experimental week to reduce systematic differences between animal batches. Normality was assessed using the Shapiro–Wilk test. Group comparisons were conducted using Student’s unpaired t-test and the Mann-Whitney U test, as appropriate. Specific analysis of SVRI changes (baseline vs. end of experiment) in the control group was performed using a paired Student’s t-test. Hemodynamic, respiratory, and metabolic parameters were analyzed using a repeated-measures mixed-model analysis. To assess differences in change from time of intervention (Δ) between groups at specific time points, SAP, SVRI, MAP, SV, HR, and CI were analyzed using multiple Student’s unpaired t-tests or Mann-Whitney U tests, and p-values were corrected using the Holm-Šídák method. Hypoglycemic events were analyzed using Fisher’s exact test. Survival curves were compared using the Log-rank (Mantel-Cox) test. Descriptive statistics are reported as mean ± SD for normally distributed data or median (interquartile range [IQR]) for non-normal distributions. Statistical significance was set at *p* < 0.05. A power analysis was performed using https://www.powercalc.ca/. To strictly adhere to the 3R principles (Reduction) by minimizing animal use, group sizes were determined using power calculations to detect clinically relevant differences in SAP at a single time point during the treatment phase. Based on previous experimental data [26], demonstrating an SAP difference of 29 mmHg (SD 10.2) between IM AVP and controls, a sample size of *n* = 4 was required (independent t-test, 1-β = 0.8, α = 0.05), and conservatively set to *n* = 5. Similarly, for the IV terlipressin group, previous data [8] indicated an expected SAP difference of approximately 60 mmHg. Assuming an SD of 10, a sample size of *n* = 3 was required (independent t-test, 1-β = 0.8, α = 0.05), which we harmonized to *n* = 5. As no prior data were available for IM terlipressin, we standardized the sample size to *n* = 5 across all groups to ensure conservative statistical power and consistency. The slightly larger control group (*n* = 7) was a strict logistical consequence of the block randomization design.

## Results

Mean hemorrhage volume was 745 (250.8) mL, representing 18.3 (6.1) % of the estimated total blood volume. Mean SAP at the end of the hemorrhagic phase was 59.8 (14.8) mmHg. Two animals died in the control group, compared with three treated with IM AVP and one in the IM terlipressin group. No animals died in the IV terlipressin group. The difference in mortality was not statistically significant (χ2 = 6.453, df = 3, *p* = 0.09). One animal was excluded due to intercurrent illness. Median core temperature at baseline was 38.3 ˚C (IQR 37.9; 38.7) and increased to 40.2 ˚C (IQR 39.7; 40.5) by the end of the experiment. The lowest recorded body temperature across all animals was 37.4 ˚C.

### Hemodynamics

**Fig. 2:**
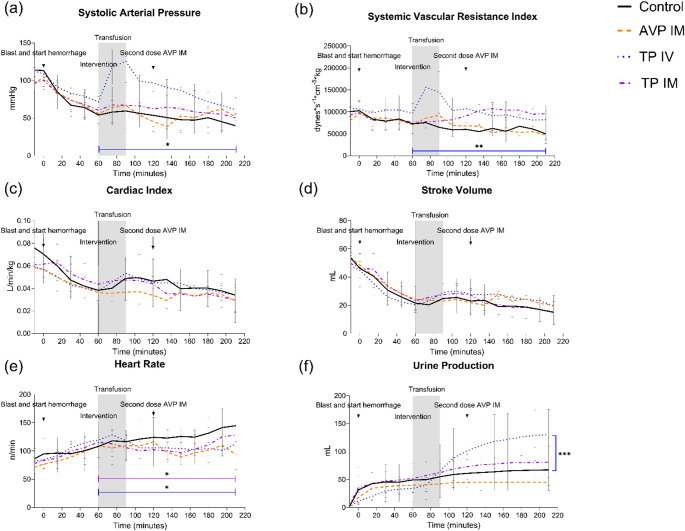
Hemodynamic effects. Systolic arterial pressure (SAP) (**a**) and systemic vascular resistance index (SVRI) (**b**) across treatment groups. IV terlipressin induced a rapid and significant increase in both SAP and SVRI within 15 min compared with controls, whereas IM AVP and IM terlipressin showed no meaningful hemodynamic effect. Cardiac index (**c**), stroke volume (**d**), and heart rate (**e**) remained largely unchanged across groups. IV terlipressin significantly increased urine output (**f**). Abbreviations: AVP, arginine vasopressin; IM, intramuscular; IV, intravenous; SAP, systolic arterial pressure; SVRI, systemic vascular resistance index. *p < 0.05, **p < 0.01

IV terlipressin significantly increased SAP compared to time of administration, difference of mean to controls: 24.4 mmHg (CI 6.8; 42.1, *p* = 0.01) (Fig. 2a). The increase in SAP following IV terlipressin was significant within 15 min after administration (*p* = 0.005). In contrast, SAP remained unaffected by administration of IM AVP and IM terlipressin compared to time of administration, difference of means: -5.6 mmHg (CI -22.5; 11.3, *p* = 0.5) respectively 5.1 mmHg (CI -11.6; 21.8, *p* = 0.51)(Fig. 2a). Additionally, IV terlipressin significantly increased MAP after 15 min by a mean of 44.2 (16.1) mmHg, compared to 3.2 (11.1) mmHg in controls (*p* = 0.001). The effects on MAP persisted for 30 min, and were no longer significant 45 min after administration, *p* = 0.06. Neither IM AVP nor IM terlipressin affected MAP, difference of means: -6.7 mmHg (CI -19.1; 5.7, *p* = 0.26), respectively 7.9 mmHg (CI -10.5; 26.3, *p* = 0.36). SVRI increased following administration of IV terlipressin compared to controls, difference of means: 44,593 dynes*s/cm^− 5^*kg (CI -17556; 71630, *p* = 0.004) (Fig. 2b). The increase in SVRI following IV terlipressin was significant within 15 min following administration (*p* < 0.001). SVRI remained unaffected by administration of IM AVP and IM terlipressin, difference of means: 11,505 dynes*s/cm^− 5^*kg (CI -11915; 34924, *p* = 0.3), respectively 20,748 dynes*s/cm^− 5^*kg (CI -5530; 47027, *p* = 0.11). In controls, SVRI decreased by the end of the experiment compared to baseline (*p* = 0.01), and SAP decreased from a baseline median of 124.1 mmHg (IQR 92.0; 127.9) compared to 34.0 mmHg (IQR 26.8; 55.8) at the end of the experiment. CI remained unaffected in all groups after administration compared to controls, difference of means: IM AVP − 0.009 L/min/kg (CI -0.03; 0.0082, *p* = 0.26), IV terlipressin 0.002 L/min/kg (CI -0.01; 0.02, *p* = 0.77), IM terlipressin 0.002 L/min/kg (CI -0.02; 0.002, *p* = 0.82), (Fig. 2c). SV was unaffected by treatment comapred to controls, difference of means: AVP IM -0.5 mL (CI -10.7; 9.6, *p* = 0.91), IV terlipressin 5.8 mL (CI -2.0; 13.7, *p* = 0.13), respectively IM terlipressin 4.3 mL (CI -5.9; 14.5, *p* = 0.37) (Fig. 2d). Heart rate decreased after administration of IV terlipressin and IM terlipressin compared to controls, difference of means: -18.4/min (CI -32.4; 4.3, *p* = 0.02), respectively − 20.0/min (CI -38.8; 1.3, *p* = 0.04) (Fig. 2e). However, administration of IM AVP showed no effect on HR compared to controls, with a difference of means of -13.1/min (CI − 41.4; 15.3, *p* = 0.33) (Fig. 2e). Central venous pressure remained unaffected in all groups after administration compared to controls, differences of mean: IM AVP − 0.9 mmHg (CI -7.9; 6.1, *p* = 0.77), IV terlipressin − 2.9 mmHg (CI -8.9; 3.0, *p* = 0.3), and IM terlipressin − 2.0 mmHg (CI -8.4; 4.3, *p* = 0.49). Pulmonary wedge pressure remained unaffected after administration compared to controls, difference of means: IV terlipressin − 0.7 mmHg (CI -2.2; 0.8, *p* = 0.31), and IM terlipressin 0.3 mmHg (CI -1.5; 2.1, *p* = 0.72). Pulmonary wedge pressure remained unaffected by IM AVP when compared to the time of administration (*p* = 0.86). Intravenous terlipressin increased urine output at the end of the experiment compared to controls, 0.6 mL/kg/h (0.19) vs. 0.12 mL/kg/h (0.14), *p* = 0.001, (Fig. 2f). Urine output at the end of the experiment was unaffected by IM AVP and IM terlipressin when compared to controls, 0.04 mL/kg/h (0.02), *p* = 0.2, respectively 0.18 mL/kg/h (0.15), *p* = 0.51, (Fig. 2f).

### Respiration

**Fig. 3:**
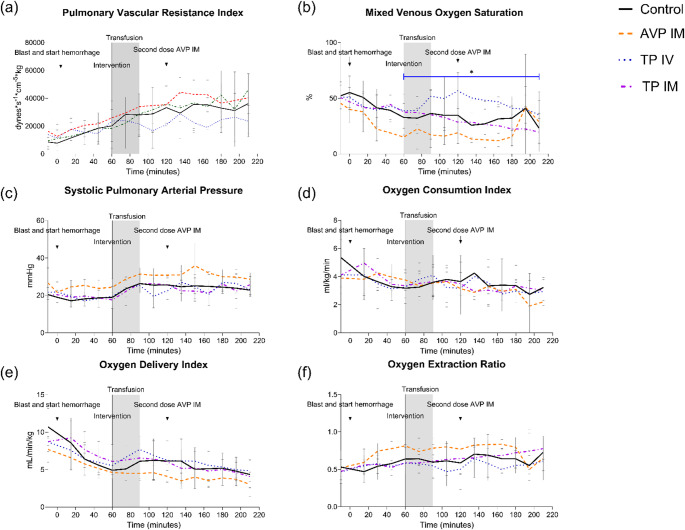
Respiratory and oxygenation parameters. Pulmonary vascular resistance index (PVRI) (**a**) did not differ between groups. Mixed venous oxygen saturation (SvO₂) (**b**) was significantly higher with IV terlipressin compared with controls, with no effect observed after IM AVP or IM terlipressin. Systolic pulmonary arterial pressure (**c**), oxygen consumption index (**d**), oxygen delivery index (**e**), and oxygen extraction ratio (**f**) were similar across all treatment groups and controls. Abbreviations: AVP, arginine vasopressin; IM, intramuscular; IV, intravenous; *p < 0.05

PVRI was unaffected between all groups compared to controls, difference of means: IM AVP 5831 dynes*s*cm^− 5^*kg (CI -10595; 22258, *p* = 0.45), IV terlipressin − 9620 dynes*s*cm^− 5^*kg (CI -24003; 4763, *p* = 0.17), and IM terlipressin − 2789 dynes*s*cm^− 5^*kg (CI -22289; 16711, *p* = 0.76) (Fig. 3a). Intravenous terlipressin increased mixed venous oxygen saturation after administration compared to controls, difference of means 14.6% (CI 2.9; 26.4, *p* = 0.02), while unaffected by IM AVP and IM terlipressin compared to time of administration, difference of means: 2.5% (CI -16.8; 21.8, *p* = 0.78) respectively − 7.9% (CI -26.2; 10.3, *p* = 0.36) (Fig. 3b). Systolic pulmonary arterial pressure remained stable when compared to time of administration, differences of means: AVP IM -0.5 mmHg (CI -4.8; 3.9, *p* = 0.8), IV terlipressin − 1.4 mmHg (CI -6.4; 3.5, *p* = 0.54), and IM terlipressin 0.5 mmHg (CI -4.4; 5.4, *p* = 0.81)(Fig. 3c). Oxygen consumption index was unaffected in all groups compared to controls: differences of means: IM AVP − 0.2 mL/min/kg (CI -1.8; 1.3, *p* = 0.74), IV terlipressin 0.005 mL/min/kg (CI -1.5; 1.5, *p* = 0.99), and IM terlipressin 0.3 mL/min/kg (CI -1.3; 1.8, *p* = 0.72) (Fig. 3d). Similarly, oxygen delivery index did not differ between the groups and controls, differences of means: IM AVP − 1.4 mL/min/kg (CI -3.7; 0.9, *p* = 0.20), IV terlipressin 0.9 mL/min/kg (CI -1.3; 3.1, *p* = 0.39), and IM terlipressin 0.7 mL/min/kg (CI -2.0; 3.5, *p* = 0.58) (Fig. 3e). Extraction of oxygen was stable after intervention when compared to time of administration, differences of means: IM AVP 0.009% (CI -0.2; 0.2, *p* = 0.9), IV terlipressin − 0.09% (CI -0.2; 0.03, *p* = 0.13), and IM terlipressin 0.02% (CI -0.17; 0.2, *p* = 0.82) (Fig. 3f). Lung compliance remained unaffected in all groups after administration compared to controls: differences of mean: IM AVP 1.6 mL/cm H_2_O (CI -4.5; 7.8, *p* = 0.57), IV terlipressin 0.2 mL/cm H_2_O (CI -6.1; 6.6, *p* = 0.94), and IM terlipressin 1.3 mL/cm H_2_O (CI -7.0; 9.7, *p* = 0.73).

### Blood gases

**Fig. 4:**
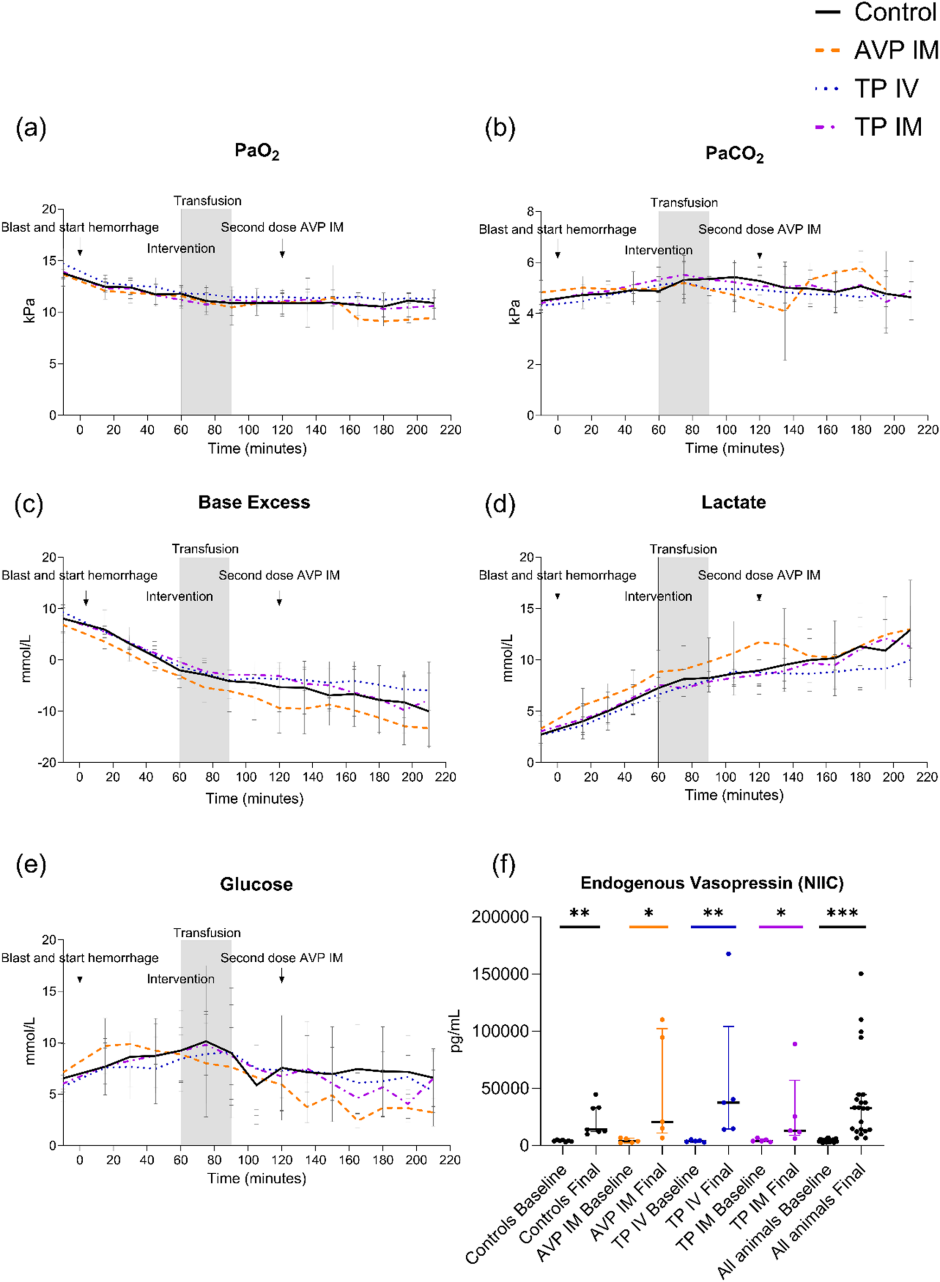
Blood gases and endogenous vasopressin. Arterial oxygen tension (**a**), carbon dioxide tension (**b**), base excess (**c**), lactate (**d**), and blood glucose (**e**) did not differ significantly following any treatment. Circulatory shock resulted in a significant increase in median endogenous vasopressin (vasopressin-NIIC) across all groups at the end of the experiment compared with baseline (**f**). Note: For animals that succumbed to trauma before the scheduled end of the observation period, terminal blood samples were drawn at the time of death and are included in the analysis of final endogenous vasopressin. Abbreviations: AVP, arginine vasopressin; IM, intramuscular; IV, intravenous. *p < 0.05, **p < 0.01, ***p < 0.001

pH remained unaffected in all groups after administration, compared to controls; differences in means: IM AVP − 0.03 (CI -0.13; 0.06, *p* = 0.41), IV terlipressin 0.06 (CI -0.01; 0.1, *p* = 0.09), and IM terlipressin 0.03 (CI -0.04; 0.1, *p* = 0.35). No differences were observed in PaO_2_ levels, differences of means: IM AVP − 0.1 kPa (CI -1.7; 1.5, *p* = 0.89), IV terlipressin 0.6 kPa (CI -0.4; 1.7, *p* = 0.2), and IM terlipressin 0.06 kPa (CI -1.1; 1.2, *p* = 0.91) (Fig. 4a). Similarly, PaCO_2_ was unaffected by the interventions, differences of means: IM AVP − 0.5 kPa (CI -1.3; 0.3, *p* = 0.21), IV terlipressin − 0.2 kPa (CI -0.9; 0.4, *p* = 0.46), and IM terlipressin 0.02 kPa (CI -0.9; 0.9, *p* = 0.97) (Fig. 4b). Base excess decreased throughout the experiment but did not differ between groups and controls, differences of means: IM AVP − 3.5 mmol/L (CI -9.5; 2.4, *p* = 0.21), IV terlipressin 2.3 mmol/L (CI -2.8; 7.5, *p* = 0.34), and IM terlipressin 1.6 mmol/L (CI -4.3; 7.5, *p* = 0.56) (Fig. 4c). Lactate levels increased throughout the experiment but did not differ compared to controls, differences of means: IM AVP 2.1 mmol/L (CI -1.4; 5.6, *p* = 0.21), IV terlipressin − 1.2 mmol/L (CI -3.8; 1.4, *p* = 0.31), and IM terlipressin − 0.6 mmol/L (CI -3.5; 2.2, *p* = 0.64) (Fig. 4d). Additionally, there were no differences in sodium levels compared to controls, differences of means: IM AVP 1.0 mmol/L (CI -0.5; 2.5, *p* = 0.16), IV terlipressin 1.2 mmol/L (CI -1.0; 3.3, *p* = 0.25), and IM terlipressin 0.4 mmol/L (CI -1.4; 2.1, *p* = 0.67). Potassium remained unaffected compared to controls, differences of means: IM AVP 0.5 mmol/L (CI -0.8; 1.7, *p* = 0.44), IV terlipressin − 0.5 mmol/L (CI -1.9; 0.6, *p* = 0.28), and IM terlipressin − 0.1 mmol/L (CI -1.4; 1.3, *p* = 0.89). Glucose did not differ between groups and controls, differences of means: IM AVP − 2.3 mmol/L (CI -7.6; 3.0, *p* = 0.35), IV terlipressin − 0.1 mmol/L (CI -5.6; 5.4, *p* = 0.96), and IM terlipressin − 0.6 mmol/L (CI -5.7; 4.6, *p* = 0.8) (Fig. 4e). In total, 12 animals became hypoglycemic (< 4.0 mmol/L) and required glucose substitution, 2 of controls, 4 of IM AVP, 3 of IV terlipressin, and 3 of IM terlipressin. There were no differences between groups in the number of hypoglycemic animals, IM AVP vs. controls (*p* = 0.24), IV terlipressin vs. controls (*p* = 0.56), and IM terlipressin vs. controls (*p* = 0.56). Moreover, there were no differences in the volume of glucose required to treat hypoglycemic animals, controls median 0 mL (IQR 0; 30), IM AVP mean 72 mL (45.5), *p* = 0.16, IV terlipressin mean 36 mL (39.1), *p* = 0.43, and IM terlipressin mean 42 mL (50.2), *p* = 0.43.

### Endogenous Vasopressin-NIIC

The median baseline Vasopressin-NIIC levels in all animals were 3894 pg/mL (IQR 3204; 5127), and increased by the end of the experiment in response to hemorrhagic shock to 14673 pg/mL (IQR 11977; 38976), *p* < 0.0001 (Fig. 4f). Vasopressin-NIIC levels were increased from baseline vs. the end of the experiment: controls 4008 pg/mL (IQR 3627; 4986) vs. 13996 pg/mL (IQR 12234; 33289), *p* = 0.0006; IM AVP 3773 pg/mL (IQR 2427; 6390) vs. 20799 pg/mL (IQR 10838; 102362, *p* = 0.02; IV terlipressin 3907 pg/mL (IQR 2966; 4860) vs. 37518 pg/mL (IQR 14400; 104095), *p* = 0.008; IM terlipressin 3881 pg/mL (IQR 3497; 5970) vs. 13032 pg/mL(IQR 8971; 57139), *p* = 0.02 (Fig. 4f). There was no difference in Vasopressin-NIIC levels between controls vs.: IM AVP (*p* = 0.5), IV terlipressin (*p* = 0.15), and IM terlipressin (*p* = 0.76).

### Systemic absorption vs. hemodynamic effects of exogenous AVP

The median baseline level of exogenous AVP in the IM AVP group was 18.8 pg/mL (IQR 16.2; 25.4). Analysis of the five animals in the IM AVP group revealed four distinct response patterns: (i) non-absorbers (*n* = 2), in which peak serum levels remained low (25.4 and 47.3 pg/mL), suggesting perfusion failure at the injection site; (ii) uncoupled responder (*n* = 1), who achieved increased systemic absorption (82.2 pg/mL) but failed to mount a hemodynamic response and died before study completion; (iii) partial responder (*n* = 1), with one animal demonstrating both high absorption (214.7 pg/mL) and a correlated hemodynamic response, with SAP temporarily rising from 71 to 102 mmHg, yet dying 75 min after intervention began; (iv) responder (*n* = 1), who achieved both increased systemic uptake (184.1 pg/mL) and a sustained hemodynamic response, with SAP increasing from 68 mmHg to 114 mmHg, and survived to the end of the experiment.

## Discussion

In this study, we demonstrate that IV terlipressin combined with whole blood transfusion increased SAP, SVRI, and SvO₂, whereas IM AVP and IM terlipressin produced only limited or inconsistent effects. To our knowledge, this represents the first evaluation of vasopressin-based therapy within the complex pathophysiology of combined blast injury and hemorrhagic shock. While our previous research supports the use of IM AVP in isolated hemorrhage [26], we demonstrate a distinct physiological divergence following blast trauma. These findings challenge the role of IM vasopressin as a universally effective strategy for hemodynamic stabilization in blast-associated hemorrhagic shock.

Between 2007 and 2017, explosive mechanisms accounted for 77.3% of reported injuries among U.S. service members, and improvised explosive devices were responsible of 60.4% of total casualties [14]. Primary blast injuries arise from rapidly propagating overpressure waves, which preferentially damage air-tissue interfaces and generate shear stresses across tissues of differing density. This extreme mechanical insult triggers the release of damage-associated molecular patterns, thereby activating complement pathways and driving a systemic inflammatory response [31, 32]. Thoracic blast exposure further induces vagally mediated reflex hypotension, with heightened parasympathetic tone blunting vasoconstrictive responses [20]. In addition, both experimental and clinical studies demonstrate increased nitric oxide after blast injury [19, 23], a mechanism likely contributing to post-blast vasoplegia and reduced systemic vascular resistance [33].

Our findings are consistent with this pathophysiologic framework. Animals in the control group exhibited a decline in SVRI despite ongoing shock, indicating vasoplegia in addition to hypovolemia. Moreover, several animals developed hemodynamic collapse disproportionate to blood loss, underscoring that circulatory failure after blast trauma cannot be explained by hemorrhage alone. Notably, one control animal lost only 3.5% of its estimated total blood volume, which was subsequently fully auto-transfused, yet died from refractory shock. Together, these observations support the concept that molecular and neurohumoral dysregulation, rather than volume loss per se, is a primary driver of cardiovascular instability following blast trauma.

Vasopressin mediates its hemodynamic effects primarily through activation of V₁-receptors on vascular smooth muscle cells, producing potent vasoconstriction and preferential redistribution of blood flow to vital organs [4]. Vasopressin analogues differ substantially in receptor affinity and pharmacokinetics. Terlipressin has approximately 2.2-fold higher V₁-receptor affinity than AVP and a markedly longer plasma half-life, resulting in more sustained vasoconstriction and prolonged hemodynamic effects [4, 5]. Species-specific factors further influence pharmacodynamics; although the porcine endogenous hormone is lysine vasopressin, human AVP remains bioactive at porcine vasopressin receptors, allowing discrimination between endogenous and exogenous hormone effects in this model [34].

Within 15 min of administration, IV terlipressin combined with whole blood transfusion increased SVRI compared with controls, consistent with V₁-receptor–mediated vasoconstriction [4]. This increase in vascular tone was accompanied by a corresponding rise in SAP, while respiratory parameters remained stable across groups. In contrast, despite prior evidence that IM AVP is systemically absorbed and hemodynamically effective in isolated hemorrhagic shock [26], this response was absent or markedly attenuated in several animals after blast injury. Blast trauma induces pronounced oxidative and nitrative stress [18, 24], including peroxynitrite formation [25, 35]. Peroxynitrite has been shown to transiently impair V₁-receptor function [22], implicating receptor hyporesponsiveness as a plausible contributing mechanism. In addition, reduced IM drug absorption was observed in some animals, consistent with impaired perfusion-dependent uptake during shock [11, 12, 26], potentially exacerbated by blast-induced microcirculatory disruption [21]. Together, these factors, variable IM absorption and transient V₁-receptor dysfunction, likely underlie the limited and inconsistent efficacy of IM vasopressin analogues in blast-associated shock. Consequently, the disproportionately high mortality observed in the IM AVP group reflects the synergistic severity of the blast-hemorrhage phenotype left unmitigated by this failing administration route, rather than the volume of blood loss alone. By contrast, IV terlipressin, with higher receptor affinity, prolonged duration of action, and complete systemic bioavailability, provided reliable and sustained hemodynamic stabilization under these conditions.

AVP typically ranges from 1 to 5 pg/mL in healthy humans, whereas it increases to 46.8 pg/mL in severe multitrauma and 507 pg/mL in transfusion-dependent hemorrhagic shock [36, 37]. In the present study, with highly variable intramuscular uptake, we observed peak AVP values up to 214.7 pg/mL, which falls within an adequate and clinically relevant range given the trauma severity. However, this must be interpreted with the caveat that our sampling protocol was not designed for high-resolution pharmacokinetic profiling; therefore, it may not represent the true peak plasma concentrations. Regarding the terlipressin cohorts, the 2 mg dose was selected as it represents the standard intravenous bolus for the management of acute variceal hemorrhage [38].

Previous studies have reported variable effects of vasopressin on CO, likely reflecting dose dependency and differences in underlying trauma physiology [4]. In the present model, IV terlipressin preserved CI despite a marked increase in SVRI, indicating maintained forward flow in the setting of increased vascular tone. Beyond its systemic effects, vasopressin also modulates renal hemodynamics by preferentially constricting efferent arterioles, thereby increasing glomerular filtration pressure and improving renal perfusion. Clinically, this mechanism has been associated with a reduced incidence of acute kidney injury and decreased need for renal replacement therapy in distributive shock [39, 40]. Consistent with these observations, IV terlipressin increased urine output to 0.6 mL/kg/h at study completion, compared with 0.1 mL/kg/h in controls. The elevated diuresis observed here likely reflects hemodynamic stabilization and restoration of renal perfusion rather than a direct renal effect of terlipressin.

This study has some limitations to be reported. First, regarding translational applicability, the highly controlled laboratory setting excludes the complex variables of prehospital and tactical environments. Furthermore, species-specific vasopressin heterogeneity, specifically the distinction between porcine lysine vasopressin and human arginine vasopressin, may influence comparative pharmacokinetics and potency. Regarding the experimental design, observations were limited to the acute phase. Ethical constraints on sample size reduce statistical power and increase the risk of type II errors. The IM terlipressin dose was limited by injection-volume constraints, which may have led us to underestimate its maximal hemodynamic effect. Finally, while our protocol intentionally restricted standard supportive measures, such as early limited crystalloid administration and continuous active warming, to strictly isolate the specific pharmacological effects of the vasopressin analogues, this experimental design is not intended to replace the multifaceted interventions crucial to real-world trauma resuscitation.

## Conclusion

In blast-associated hemorrhagic shock, with profound hemodynamic instability disproportionate to blood loss, IV terlipressin provided consistent hemodynamic stabilization, whereas IM vasopressin analogues proved unreliable. Specifically, IM AVP demonstrated highly heterogeneous pharmacokinetics and clinical efficacy, highlighting a critical limitation of the intramuscular route in the context of blast pathophysiology.

## Data Availability

Data are available through the corresponding author.

## Abbreviations

AVP: Arginine vasopressin
CI: Cardiac index; confidence interval (used in two senses)
CO: Cardiac output
HR: Heart rate
IM: Intramuscular
IQR: Interquartile range
IV: Intravenous
MAP: Mean arterial pressure
NaCl: Sodium chloride
PVRI: Pulmonary vascular resistance index
SAP: Systolic arterial pressure
SD: Standard deviation
SV: Stroke volume
SVRI: Systemic vascular resistance index
Vasopressin-NIIC: Vasopressin-neurophysin 2-copeptin
